# Palliative oncology and palliative care

**DOI:** 10.1002/1878-0261.13278

**Published:** 2022-08-12

**Authors:** Peter Strang

**Affiliations:** ^1^ Department of Oncology‐Pathology, Karolinska Institutet, Regional Cancer Centre in Stockholm – Gotland, and R & D Department Stockholm's Sjukhem Foundation Sweden

**Keywords:** early integration, oncology, palliative care services, personalized medicine, quality‐of‐life, translational research

## Abstract

New therapeutic approaches can produce promising results even in severely ill cancer patients. But they also pose new challenges with respect to prognostication, as patients who were once not eligible for treatment, due to age or comorbidities, now are. Palliative oncology constitutes a major part of oncological care, with life prolongation and quality of life as its main goals. Palliative care specialists are experts in symptom control and psychosocial and existential support, and the integration of their expertise early on in patient care can prolong survival. In this article, I discuss the need to integrate specialist palliative care into early cancer treatment plans to achieve quality of life for patients. I also discuss the ways in which palliative care specialists balance the benefits of novel treatments against their adverse effects for patients, particularly for the elderly, the frail and those in advance stages of disease. I highlight the need to ensure equal access to palliative care to improve cancer patients' quality of life but also why futile, burdensome treatments should be avoided especially in the frail, elderly patients. Further, I discuss benefits and problems related to nutritional support in patients with cachexia and exemplify why translational research is needed to link basic research with clinical oncology and effective symptom control.

## Graphical abstract

Palliative oncology constitutes a major part of oncological care with life prolongation and quality of life as its main goals. Novel treatment opportunities with targeted therapies and other immunotherapies show promising results but must be balanced against a new type of adverse effects. This policy article explores how integration of palliative oncology and palliative care might facilitate treatment decisions and improve symptom control in patients with cancer.

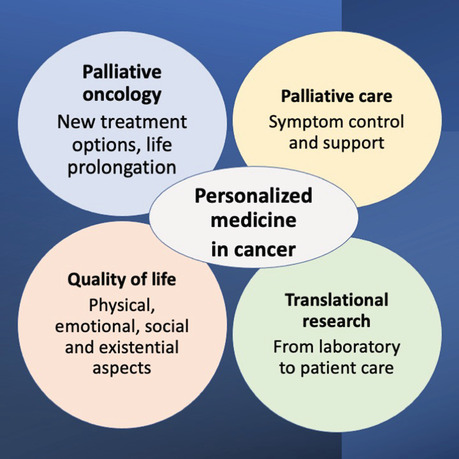

**Abbreviations**
APHC: advanced palliative home care
ESPEN: The European Society for Clinical Nutrition and Metabolism
ICU: intensive care unit
PROM: patient‐reported outcome measures
SBRT: stereotactic body radiotherapy
SPC: specialized palliative care

## 1 Introduction

Even today, despite prevention efforts and treatment, 2.6 million people in the 27 countries of the EU are diagnosed every year with cancer [1]. Although the age‐standardized mortality rate in EU countries is declining [2, 3], the absolute number is expected to increase due to an aging population, as cancer is an aging‐related disease. Today, 1.2 million people die from cancer in the EU every year [1]. However, major progress had been made in all fields of oncology, from preclinical to clinical research, and from prevention to treatment. As a result, the European Academy of Cancer Sciences (EACS), in collaboration with a number of European cancer organizations, published advice in 2020 concerning areas of research to prioritize, to ensure a balanced research portfolio, and provided recommendations for how to meet the key targets set out [2]. A key goal of the EACS and its partners is to achieve a 10 years survival period for 75% of the adult population diagnosed with cancer in the EU by 2030 [2]. Even were such a high goal achieved, the proportion of patients with incurable cancer would remain considerable.

In this article, at the invitation of the EACS, I review how palliative oncology, patient nutrition and treating cachexia, have undergone changes and improvements in cancer care and in geriatric oncology, as this is also a new opportunity for elderly patients. Cancer cachexia is central to palliative oncology, as it decreases patients´ quality of life, it is associated with poor responses to antitumor therapy, as well as with decreased survival.

I discuss the need to integrate oncology, palliative care, and cost of care early in patient treatment, as an integration might be beneficial to improve symptom control and to balance opportunities and risks associated with new treatments. Further I discuss aspects of personalized medicine and patient‐reported outcomes (PROMs) in order to improve quality of life. Finally, I exemplify why translational research has a potential also to improve symptom control and I end by describing why equal access to palliative care should be a priority.

## 2 Palliative oncology—a major part of oncological treatment

Traditionally, palliative care concerns treating the dying, but the WHO definition of palliative care (dating back to 1990), is as follows: Palliative care is [4]

The active total care of patients whose disease is not responsive to curative treatment. Control of pain, of other symptoms, and of psychological, social and spiritual problems is paramount. The goal of palliative care is achievement of the best possible quality of life for patients and their families. Many aspects of palliative care are also applicable earlier in the course of the illness, in conjunction with anti‐cancer treatment.

This was a visionary statement and in the WHO definition of palliative care in 2002, the following statement was added: “[Palliative care] enhances quality of life, promotes dignity and comfort, and may also positively influence the course of illness”.

Considering the definition above, there is no hard dividing line between palliative oncology and palliative care, although this is seldom recognized. A more conscious integration of palliative oncology and palliative care measures is more important than ever, as new treatments are available and extended also to elderly and frail persons, i.e., new groups who might benefit from the treatments but who also are more vulnerable.

In the past decades, considerable progress has been made in the treatment of metastatic cancer. In the early 1980s, a distant metastasis was, with very few exceptions, a sign of an incurable disease. Today, surgical metastasectomy (Box 1) is an option in selected cases [5], and stereotactic body radiotherapy (SBRT) (Box 1) has been used to treat oligometastatic spine metastases (Box 1) [6], with a curative intent. In general, the dichotomized view of curable and incurable cancer is being replaced by a view that is characterized by a continuum, in which cases with stable oligometastases represent an intermediary state [7, 8, 9].

Box 1 **Glossary**

APHC: advanced palliative home care.

Cancer cachexia: systemic inflammation resulting in a multifactorial syndrome defined by an ongoing loss of skeletal muscle mass (with or without loss of fat mass) that cannot be fully reversed by conventional nutritional support and leads to progressive functional impairment and poor prognosis.

ICU: intensive care unit.

Metastasectomy: to remove a single or a few metastases by surgery or stereotactic body radiotherapy (SBRT), with a curative intent.

Oligometastasis: a clinical state of metastasis that refers to restricted tumor metastatic capacity in a patient with one or only few metastases that can be treated with curative intent.

Performance status (PS)/performance index is used in all oncological care. PS 3–4 means that the patient is ambulatory < 50% of time and nursing care is needed, or, the patient is bedridden.

PROM: patient‐reported outcome measures, i.e., self‐assessments.

SPC: specialized palliative care.

SBRT (stereotactic body radiotherapy): external beam radiotherapy used to deliver a high dose of radiation very precisely to an extracranial target within the body, as a single dose or a small number of fractions.

Targeted therapy: molecules targeting specific enzymes, growth factor receptors, and signal transducers, thereby interfering with a variety of oncogenic cellular processes, with the aim of life‐prolongation.

Despite these advances, a substantial proportion of cancer patients will develop an incurable, disseminated disease, with life‐prolongation and quality of life forming their main treatment goals. Among the new therapeutic options that prolong life in cancer patients, even for those with advanced cancer, are the targeted therapies and other immunotherapies [10, 11]. Today, targeted therapies are also used as first‐line therapies, for example, in the treatment of metastatic colorectal cancer and malignant melanomas, the latter being among the most susceptible cancers to immunotherapies [10, 12, 13]. In contrast to curable patients, those in the palliative stages of care will be treated for long periods, in many cases for several years, and this trend is increasing with new upcoming treatments [10, 12, 13]. As such, most of cancer treatment is, in fact, directed toward palliative oncology.

However, recent improvements in cancer treatment and care that prolong life pose new challenges with respect to prognostication and treatment intensity. In previous years, very few patients with metastatic cancer would have been treated in intensive care units (ICUs), now that picture is changing [14]. There are cases where well‐founded decisions are being made to initiate intensive cancer treatment, despite patients having disseminated disease, which presuppose that the resulting adverse effects will be addressed, even where ICU treatment might be needed in selected cases.

## 3 New treatment options, new challenges

How cancer is treated has changed very rapidly during the past 10–15 years, with targeted therapies and other immunotherapies being used to treat even very advanced disease. These innovative treatment options enable multiple sequential treatments with different drugs and they prolong life, which in turn places new demands on cancer care. Today, many patients receive their cancer therapy at cancer centres in hospitals. Meanwhile the daily care, including symptom control, psychosocial support, and the management of potentially harmful adverse effects, is often provided by skilful palliative home care teams or other health care services [15].

These palliative care teams are likely to be very familiar with the usual side‐effects caused by traditional chemotherapy treatments, such as nausea, vomiting, and hair loss. However, they may have less experience of the side‐effects that are regularly caused by targeted therapies and immunotherapies. As examples, immune checkpoint inhibitors (ICIs) are known to produce mild to severe endocrinopathies in the form of thyreoiditis, hypophysitis, adrenal insufficiency, and diabetes mellitus [16]. They can also cause severe hematological, neurological, musculoskeletal, and cardiovascular adverse effects, which may be life‐threatening [17, 18, 19, 20, 21]. The early recognition and management of such adverse effects are, therefore, highly important. Most of these adverse effects are manageable with systemic glucocorticoids, but the indications and dosing require specialist knowledge [20]. For this reason, the palliative care physician should consult the oncologist in charge of a patient's treatment [20]. Today, many clinical trials allow for the use of oral prednisone of up to 10 mg daily [20], but this may differ from trial to trial.

## 4 Cachexia and nutritional aspects in the cancer continuum

In this new situation where the lives of cancer patients can be prolonged for long periods, the nutritional aspects of their care are of great importance. The risks of developing malnutrition and cancer cachexia (Box 1) is much higher in patients receiving palliative treatment, compared with those with newly diagnosed, non‐metastatic cancers [22]. Whereas patients with curable cancers seldom manifest cachexia, its prevalence in those with incurable cancer, depending on diagnosis and stage, can range from a few percent to as high as 50–80% in those at advanced disease stages [22]. This is important for several reasons: cancer cachexia, which is characterized by weight loss, muscle wasting, anorexia and inflammation, negatively affects patients' quality of life [22, 23]. Moreover, it reduces the effectiveness of cancer therapies and increases their toxicities [23]. Although a commonly accepted definition for cachexia is lacking, many scientists classify it into three main groups: precachexia, cachexia, and refractory cachexia (Box 2), but not all patients experience all of these stages [24]. Cachexia is associated with poor survival prospects, which is especially true for patients with refractory cachexia [22], and cachexia itself accounts for 20–30% of all cancer‐related deaths [23, 25]. Primary cachexia with an imbalance in metabolic regulation should, of course, be distinguished from malnutrition, which is sometimes referred to as secondary cachexia and is caused by eating difficulties that are related to reversible causes, such as pain and obstructions.

Box 2 Pre‐cachexia: weight loss ≤ 5%. Anorexia and metabolic change.

Cachexia: weight loss > 5% or BMI <20 and weight loss >2% or sarcopenia and weight loss > 2%. Often reduced food intake/systemic inflammation.

Refractory cachexia: variable degree of cachexia. Cancer disease both procatabolic and not responsive to anticancer treatment. Low performance score, less than 3 months expected survival

For these reasons, nutritional strategies should be in place in the early phases of treating a patient with disseminated disease, in order to promote their quality of life and to facilitate cancer treatments. ESPEN (the European Society for Clinical Nutrition and Metabolism) has recently updated their recommendations for different stages and treatment situations in the cancer trajectory [26]. ESPEN also recommends early nutritional intervention; by contrast, nutritional interventions are seldom beneficial during the last weeks of life [26]. General recommendations, irrespective of disease stage, include screening and assessing cancer patients for malnutrition; assessing their energy requirements, and making nutrition and life‐style interventions, including dietary advice, exercise to avoid muscle wasting, and the use of pharmaconutrients and pharmacological agents, when needed [26].

## 5 Elderly cancer patients

Many elderly patients, who would not have been candidates for palliative oncological treatments in the past, are now increasingly being treated. As a result, oncologists now also need to have basic knowledge in geriatric oncology, especially when treating disseminated cases, and need to understand how frailty affects the adverse effects of cancer treatment, as well as its outcomes [27]. Frailty is defined as an age‐related condition that is characterized by decreased physiological reserves, loss of adaptive capacity, and an increased vulnerability to stressors [27]. In oncology, frailty is associated with more adverse effects, poorer survival prospects, and a higher risk of not completing a planned therapy [27]. Frailty is also associated with comorbidities but can exist independently of these [27]. Thus, the oncologist is faced with a new challenge: the option to prescribe meaningful, life‐prolonging treatment to patient groups who were previously not eligible, balanced against the need to recognize the risks and contraindications. These more recent treatment options and new treatment modalities both need to be carefully monitored in the frail population.

## 6 Benefits of early integration

Palliative care is in some contexts seen as a passive care but as early as 1990, the WHO highlighted its role in the early care of cancer patients. Moreover, the WHO's definition of palliative care in 2002 highlighted its ability to “positively influence the course of illness”. Indeed, this was proven in a well‐received publication by Temel et al. [28], who showed that the early integration of palliative care translated into better quality of life, fewer depressive symptoms, and to statistically prolonged median survival, despite the fact that patients randomized to both cancer care and palliative care received aggressive end‐of‐life care to a lesser extent. Still, the optimal palliative care approaches to employ in early integration remain to be defined for different diagnoses and situations, as data on these issues is still scarce and the absolute benefits of palliative care are insufficiently known [29, 30].

As already stated in the WHO definition, the goal of palliative care is to provide the best possible quality of life for patients and their families. How is this achieved? And to what cost? Besides the burden of treating adverse effects, studies also show that the costs of a cancer patient's last year of life are significant and constitute about a third of the costs for cancer care [31]. However, the most expensive and aggressive treatment is not necessarily the optimal choice for every patient in a palliative stage of care [28]. As such, adherence to evidence‐based guidelines is of great importance, to reduce the use of costly and unproven cancer treatments. The American Society of Clinical Oncology (ASCO) proposes five key elements to improve care and still reduce costs [32].

One of these recommendations is relevant to palliative oncological or palliative care situations: physicians should be restrictive with anticancer treatments for patients with solid tumors who have a permanent, low performance status (3 or 4, i.e., ambulatory < 50% of time, or bedridden) (Box 1), which is not to be confused with a temporary low performance status, for example, due to an infection. This is one example of how to balance requests for new treatments with evidence‐based facts.

Today, the media are eager to report new, promising treatments and also anecdotal cases where exceptional responses have been achieved, but they seldom discuss the limitations, for example, that a specific treatment is limited to a specific diagnosis. Neither do they publish data on serious adverse effects, treatment failure or deaths due to complications [33]. This causes a situation in which patients and their families find descriptions of new, promising treatment options online and in the media, and the oncologist needs to have relevant and up‐to‐date information about the pros and cons of these treatments, based on evidence.

The integration of specialist palliative care (SPC) and oncology is also beneficial from this point of view, as palliative medicine specialists are trained to provide a balanced account of information to patients. Moreover, SPC providers can provide adequate and timely care for cancer patients in the late stages of disease within the available services. In this way, the integration of SPC with cancer care translates into fewer emergency department visits, fewer readmissions to hospitals, and fewer cases where patients die following acute hospitalization [34, 35, 36, 37].

## 7 Personalized care: quality of life and life prolongation

Traditionally, the main outcome when evaluating the effect of palliative cancer treatment, has been survival, i.e., the prospects of life prolongation. Although palliative care is often associated with the concept of ‘quality of life’ [38], the importance of life prolongation should not be underestimated. In fact, life prolongation in itself might lie at the core of quality of life for many cancer patients in a palliative stage [39, 40]. The awareness of an insecure future and the constant threat of death might make even limited life prolongation valuable [39]. In fact, patients in late palliative stages might even associate a “good doctor” with one that provides hope in the form of life prolongation [41], since life prolongation of just a few months translates into more months with one's spouse, children and grandchildren. This view of life, and the will to live for as long as possible, that is often held by severely ill cancer patients is very different from that of a healthy person in the middle of their lives. In one study of seven European countries, most of the 9344 interviewed individuals (telephone survey of a random sample of households) stated that they would prioritize quality of life over life prolongation [42]. Therefore, when planning for patients in end of life situations, the opinions of the patient themselves must be asked as they might differ from the opinions of wider society.

Besides life prolongation, the effective relief of symptoms is paramount for two reasons: (a) the relief of physical suffering is desirable and improves patient quality of life and (b) effective symptom control translates into improved existential wellbeing, e.g., in the form or reduced death anxiety, as patients associate severe pain with disease progression. Severe symptoms that are not adequately treated trigger thoughts in patients about impending death [39]. In this context, cancer‐specific treatments and symptom control should not be discussed as entirely separate issues. This is because successful cancer treatment results in a complete or partial response, or in stable disease, and often translates into fewer cancer‐related symptoms, as well [43].

In a broader context, being in a palliative stage represents a multifaceted situation in which treatment success should not only be related to life prolongation, but also to symptom burden, performance status, nutritional status, and weight loss, as they all affect quality of life [32, 38, 44]. An ASCO statement points out that while personalized care and treatment for curable disease mainly focus on the unique biological features of a patient's disease, personalized care for incurable disease also focuses on the physical, psychological, social, and existential consequences of the disease [38]. The balance between benefits and risks is especially important during the last month of life, when continued anticancer treatment might be associated with a significantly increased rate of adverse events and related mortality [45]. For this reason, the integration of oncological care and SPC is highly desirable.

Rational treatment decisions are also desirable, but a person facing his or her impending death is not rational but in a state of emotional turmoil [39, 41, 46, 47]. Anxiety, especially in the form of death anxiety, is a strong driving force for treatment requests [39, 41, 48], and it is associated with an increased likelihood of continuing chemotherapy even in the 30 days before death [49, 50]. Nevertheless, palliative chemotherapy during the last weeks of life is not associated with improved quality of life. Instead, it might have a negative impact on dying patients due to its adverse effects [51]. Palliative care specialists are trained to discuss such issues and to balance hope with the actual risks.

## 8 Quality of life and patient‐reported outcomes

As already briefly mentioned, quality of life in the form of health‐related quality of life is an important goal in palliative care. In general, quality of life is negatively affected by physical symptoms (such as pain, nausea, dyspnea, and fatigue), and by mental and psychological problems (such as anxiety, depression, and sleeping difficulties), social issues (such as family and financial worries, living arrangements) and existential or spiritual distress. Therefore, assessments of symptoms and of health‐related quality of life should be multidimensional and part of a patient's follow‐up, both in curative and palliative situations. The European Organization for Research and Treatment of Cancer (EORTC) has provided an impressive 61 questionnaires to use in such assessments [52]. Among these, there are core questionnaires, as well as diagnosis‐specific modules (e.g., for breast or lung cancer), and questionnaires for specific conditions (e.g., for cancer cachexia or spiritual wellbeing) [52]. While several of the modules have questions about typical chemotherapy adverse effects, there are, so far, no available questionnaires about the adverse effects associated with targeted therapies or immune therapies. This is a problem, as validated instruments are only valid in the very context that they were validated in. Thus, there is a need to develop validated instruments for use in these current anti‐cancer treatment regimens. So far, much effort has gone into predicting candidates for successful treatment but, obviously, there is also a need to identify those at risk of severe adverse effects, particularly in response to targeted therapies and other immunotherapies.

When making decisions about cancer care, patient‐reported outcomes (PROMs) should be taken into consideration [53, 54, 55]. The patients' perspective is important as the problems and preferences of individual patients is likely to differ. Moreover, PROMs are valid outcomes in cancer [55]. A systematic symptom assessment improves wellbeing, but also awareness of a patient's individual situation, which might translate into improved survival [56, 57]. The patient perspective should also be determined when toxicities are reported. Often, a tool called Common Terminology Criteria for Adverse Effects (CTCAE) is used by physicians to report adverse outcomes but there is also a patient‐reported outcomes version, called PRO‐CTCAE, that should also be used [58, 59].

It goes without saying that health‐related quality of life measurements are important tools for assessing cancer symptoms and toxicities and are useful on a group level. By contrast, a person's individual quality of life is largely influenced by their individual social and existential experiences and would not be covered by questionnaires.

## 9 Optimal oncological palliative care services

The organization and the integration of palliative care services are different in different countries. In Sweden, all health care services should be able to provide basic, general palliative care, but for cancer patients who often present with complex symptoms and with greater needs of care and support, specialist palliative care (SPC) is needed [15, 60]. In Sweden, SPC mainly relies on advanced palliative home care (APHC) teams that operate 24 h a day and are staffed by registered nurses, physicians, physiotherapists, occupational therapists, dieticians, and social workers. In many parts of Sweden, such APHC teams provide palliative care in parallel with the oncological care provided by hospitals, resulting in effective symptom control and satisfaction with the support offered [15]. When needed, palliative care is also provided by SPC services in hospitals. A weakness in the current Swedish health care system is the lack of consultation teams (i.e., teams of physicians and registered nurses with special competence in palliative care), that could make specialist consultations in the hospitals. Other countries have chosen different solutions, including the use of consultation teams. The important thing is that palliative care services should be integrated with oncological care in a way that creates synergies, and where each area of responsibility is well‐defined; the patient should know whom to call in different situations.

## 10 Equal access to palliative care

In a well‐functioning health care system, access to SPC should be equal for all patients with similar needs, regardless of their sex, age, or socio‐economic status. Nevertheless, studies highlight inequalities in cancer care and that the likelihood of receiving hospice care differs depending on the healthcare system. As an example, cancer patients who were younger, male, black, unmarried, and those with lower incomes were less likely to receive hospice care within the US Medicare system [61]. In a recent review, outlining disparities in hospice and palliative care access in the United States, higher socio‐economic status, and being female, were variables associated with access to palliative care [62]. Access to palliative care also differs between countries. For example, in Sweden, sex is not a determinant of palliative care, whereas age seems to be an important factor insofar as younger patients have a greater access to SPC than do older patients [37], in line with other studies [62].

It should nevertheless remain the goal of health services that SPC is offered to all cancer patients on an equal basis. This is not least because SPC services can successfully manage symptoms and acute conditions [14]. These successes translate into significantly fewer emergency room visits, fewer acute admissions to hospitals, and fewer hospital deaths [37, 63]. Since many patients prefer home as their place of death, fewer hospital deaths are also a quality of life issue [64].

## 11 Palliative care, chronic care, or supportive care?

The use of words and terms matters, as terms that are not properly understood but perceived as threatening by patients, their families and even by health care professionals, will constitute a barrier to the provision of optimal care [65, 66]. This is especially the case when patients, and even medical staff, associate palliative care with death and dying. By contrast, modern palliative care experts think it is obvious that palliative treatment also includes early palliative stages where life‐prolongation is of paramount interest [65, 66]. In a survey of medical oncologists and midlevel providers, it was evident that a considerable proportion of physicians were reluctant to refer a patient if the term palliative care was used, but more prone to do so if the term supportive care was applied instead [65].

Today, these terms are sometimes used interchangeably. Moreover, the WHO has also introduced the term “chronic”, when referring to cancer as one of four chronic diseases [67]. However, “chronic” is not a well‐defined term in medicine and should be used cautiously [68].

The goal is to optimize patient access to palliative care and to improve different outcomes [66]. Still, previous studies have shown that the term supportive care, in contrast to palliative care, is easier to accept and is associated with more and earlier admissions [69]. If palliative care is merely understood as care of the dying, despite efforts to explain the term to patients and their families, the choice of optimal terms should be carefully considered, especially when communicating with patients. From a substantive point of view, both terms are possible to use. In fact, David Hui and Eduardo Bruera define palliative care as “supportive care that focuses on patients with advanced‐stage diseases” [66, 70]. Nevertheless, the issue of which term to use remains a complex one, as the term “palliative” might constitute a barrier to early referral, while the term “supportive” might hamper acceptance and preparation for an impending death at later stages of disease.

## 12 Translational research at the interface of tumor biology and symptoms

Translational research has the potential to create synergies between preclinical, clinical and palliative care research, but unfortunately, it is seldom done, not even in central research areas. Cachexia exemplifies an interesting and urgent field for translational research, as cachexia has a substantial impact on quality of life, as well as on survival [22, 23, 24]. Moreover, cachexia is not only a question of malnutrition, which can be reversed by nutrition, but a most complex condition associated with cancer progression and premature death [71].

Recent studies indicate that certain cachexia signals promote the development of cachexia. If such signals [such as ActRIIB, histone deacetylase (HDAC)1, and STAT3] are blocked, in laboratory conditions, survival can be prolonged in animal cancer models, even though their tumors continue to grow [25, 72, 73]. A better understanding of the underlying mechanisms of this disorder would improve both the prognosis and quality of life for patients with manifest cachexia.

Bone metastases represent another area of interest for translational research. Bone metastases are associated both with pain and tumor progression and can be modeled in animals. Currently, there are both osteolytic and osteoblastic tumor models in mice, in which biological studies of bone metastases can be investigated. Certain tumors, such as oral, prostate, or pancreatic tumors, produce nerve growth factor (NGF), which has a role in neuropathic pain in metastatic cancer [74], and in the development of cachexia and tumor progression [75]. Nerves infiltrate the tumor microenvironment thereby enhancing cancer growth and metastasis, a research field sometimes referred to as nerve‐cancer cell cross‐talk [76, 77, 78]. Findings from this field need to be translated into clinical research in patients with symptoms, especially as there are anti‐NGF treatments with proven efficacy in laboratory models [79], and these models explain why drugs, typically used for neuropathic pain, e.g. gabapentin, have a pain relieving effect in certain patients with bone pain.

## 13 Conclusions and perspectives

As new successful treatment options emerge, the oncologic panorama is changing. Patients with oligometastases are to a higher degree curable and patients with disseminated disease live longer. The cancer trajectory is less foreseeable as new therapies means some major and unforeseeable responses. New patient groups, including elderly patients and cancer patients are possible candidates for treatment, at the same time futile and even hazardous treatments should be avoided, which makes prognostication even more important. Early integration should be aimed at, as such integration has a potential to improve symptom control and quality‐of‐life.

The treatments should be personalized and PROMs should have a greater impact in the decision‐making. Moreover, palliative treatments and palliative care for cancer patients should be offered, regardless of sex, age, or socio‐economic factors. In the future, translational research has a great potential to link preclinical studies with oncological outcomes and also with symptom control.

## Conflict of interest

The authors declare no conflict of interest.

## Author contributions

Peter Strang (solely) made the conception and the design and wrote the manuscript.
